# Capillary Liquid Chromatography for the Determination of Terpenes in Botanical Dietary Supplements

**DOI:** 10.3390/ph14060580

**Published:** 2021-06-17

**Authors:** Henry Daniel Ponce-Rodríguez, Jorge Verdú-Andrés, Pilar Campíns-Falcó, Rosa Herráez-Hernández

**Affiliations:** 1 MINTOTA Research Group, Departament de Química Analítica, Facultat de Química, Universitat de València, Dr. Moliner 50, 46100 Burjassot, València, Spain; Henrypon@alumni.uv.es (H.D.P.-R.); Jorge.Verdu@uv.es (J.V.-A.); pilar.campins@uv.es (P.C.-F.); 2 Departamento de Control Químico, Facultad de Química y Farmacia, Universidad Nacional Autónoma de Honduras, Ciudad Universitaria, Tegucigalpa 11101, Honduras

**Keywords:** natural products, plant materials, dietary supplements, terpenes, capillary liquid chromatography

## Abstract

Dietary supplements of botanical origin are increasingly consumed due to their content of plant constituents with potential benefits on health and wellness. Among those constituents, terpenes are gaining attention because of their diverse biological activities (anti-inflammatory, antibacterial, geroprotective, and others). While most of the existing analytical methods have focused on establishing the terpenic fingerprint of some plants, typically by gas chromatography, methods capable of quantifying representative terpenes in herbal preparations and dietary supplements with combined high sensitivity and precision, simplicity, and high throughput are still necessary. In this study, we have explored the utility of capillary liquid chromatography (CapLC) with diode array detection (DAD) for the determination of different terpenes, namely limonene, linalool, farnesene, α-pinene, and myrcene. An innovative method is proposed that can be applied to quantify the targets at concentration levels as low as 0.006 mg per gram of sample with satisfactory precision, and a total analysis time <30 min per sample. The reliability of the proposed method has been tested by analyzing different dietary supplements of botanical origin, namely three green coffee extract-based products, two fat burnings containing *Citrus aurantium* (bitter orange), and an herbal preparation containing lime and leaves of orange trees.

## 1. Introduction

Today, a wide variety of products are available intended to supplement the diet with the idea of promoting health and wellness. Dietary supplements are considered products at the interface between pharma and nutrition [1,2]. However, the regulations established for their preparation and distribution are not as strict as those set for pharmaceuticals [3]. Of particular concern are preparations that contain mixtures of plants, either in the whole form or as extracts, because they are perceived as safe for consumers due to their natural origin. Unlike homogeneous pharmaceuticals, supplements elaborated with similar ingredients may contain highly variable amounts of active compounds, depending on the plant sources and processes used during their production [4]. For these reasons, increasing attention is being paid to control the quality and efficacy of botanic dietary supplements through the analysis of their bioactive components [3,5]. In addition, adulteration and counterfeiting (such as the use of prohibited additives and incorrect botanical or geographical declaration) are frauds commonly detected in dietary supplements. Therefore, adequate analytical methods are required to detect such manipulations [6]. 

Plants are sources of several functional compounds, such as phenols, alkaloids, steroids, terpenes, and others. In particular, terpenes have been reported to exhibit diverse beneficial effects including anti-inflammatory, antimicrobial, anticarcinogenic or anti-aging [7,8,9]. Because of such properties, the levels of terpenes are gaining importance for assessing the biological activity of a variety of medicinal plants and dietary supplements [4,10]. Terpenoids are the main constituents of essential oils, and due to their volatility, they are responsible for the aroma. Some representative terpenes, such as linalool, have been proposed as biomarkers to detect adulterations and to control the safety of marketed products [11]. Due to its antimicrobial activity, limonene has been proposed as an ecological preservative in some food products [12]. 

Gas chromatography (GC) has been extensively used for the analysis of volatile terpenes in plants. The studies reported during the past years were mainly focused on establishing the terpenes profile of different vegetal species and fruit beverages [13,14,15,16,17,18,19]. Only a few works dealt with the determination of terpenes in products aimed at enhancing health. This is the case of the study reported by Mukazayire et al., who described a method for the characterization of essential oils in medicinal plants with notable antioxidant activity [20]; the investigation included some terpenic compounds. As most of those studies were aimed at establishing the volatile profile of the tested plants, GC with mass spectrometry (MS), or GC × GC-MS, often in combination with multivariate data treatment, were the analytical techniques used. Liquid chromatography (LC), on the other hand, is the predominant technique in the analysis of plants used as food and medicines, although its application to the analysis of terpenes is rare [10]. This can be explained by the fact that terpenes are volatile and thus, well suited for GC, and also because of the lack of chromophores in their structure, which can be a serious limitation considering that the levels of terpenes in this kind of samples are usually low (<1%) [4]. 

Recent progress in LC has resulted in miniaturized scale separations, such as capillary LC (CapLC) with enhanced resolution and sensitivity derived from the fact that the dispersion of the analytes during the separation is considerably reduced [21]. Miniaturized LC offers additional advantages such as lower consumption of mobile and stationary phases, and a reduction in the generation of wastes [22]. However, in the analysis of dietary supplements, CapLC has only been applied to a few compounds so far, mainly amino acids and peptides, fatty acids, and flavonoids [23]. Therefore, the potential of CapLC for the analysis of terpenes in dietary supplements remains unexplored. In a recent study, we demonstrated that CapLC is a valuable tool for estimating the content of terpenes of resins obtained from different trees. Because of the high sensitivity attained, the method could be applied to the analysis of limonene, amyrin, lupeol, and lupenone in microsamples of resins [24].

Taking advantage of its high sensitivity, in the present study we report for the first time the application of CapLC to the quantification of representative terpenes in dietary supplements. The target compounds selected were limonene, linalool, farnesene, α-pinene, and myrcene. The chemical structure and main biological effects of these compounds according to the literature are shown in Table 1. The analytes were previously extracted from the samples in methanol using an ultrasound-assisted extraction (UAE) protocol. The proposed approach has been applied to the analysis of different commercial products containing botanical species as main ingredients with a variety of claimed properties (stimulant, fat-burning, and relaxant). 

## 2. Results

### 2.1. Separation and Analytical Performance

**Figure 1:**
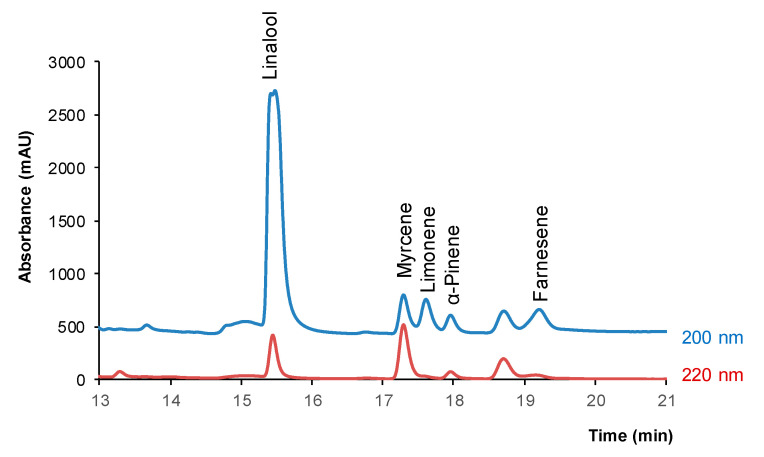
Chromatograms obtained under the selected conditions at 200 nm and 220 nm for a standard solution of the tested compounds (5 µg mL^−1^ each). For other experimental details, see text.

Different chromatographic conditions were assayed to achieve a satisfactory resolution of the analytes from other plant constituents. As it can be deduced from Table 1, the target analytes do not have polar groups (e.g., -OH or -NH_2_) in their chemical structure. As a result, they are quite hydrophobic substances; the log of their octanol-water partition coefficients ranges from 2.97 (linalool) to 7.10 (farnesene) [27]. Thus, they were expected to elute at high retention times under reversed-phase separation conditions. With this in mind, different experiments were carried out with standard solutions of the analytes and with methanolic extracts of the samples tested in order to select a gradient elution program adequate for the separation of the analytes from other plant constituents with polar groups (polyphenols, amines). Most matrix compounds were found in the first part of the chromatograms under the conditions selected (Section 4.2) whereas the analytes eluted in the 15–20 min time window, and they were satisfactorily resolved, as can be seen in Figure 1. In this figure are depicted the chromatograms obtained at 200 nm and 220 nm for a mixture of the tested terpenes (5 µg mL^−1^ each compound). Although myrcene exhibited higher absorptivity at 220 nm, the peak areas were higher for most compounds at 200 nm (detector saturation was observed for linalool at 200 nm). Therefore, 200 nm was selected as the working wavelength. The retention times are listed in Table 2.

Next, the analytical performance was evaluated by processing standard solutions of the analytes. The working concentration ranges were selected in order to obtain peak areas of about the same order for all the analytes at the working wavelength (200 nm). The results of this study are summarized in Table 2.

The linearity was evaluated for each compound by processing in duplicate five concentrations within the tested concentration range. Satisfactory linearity was observed in all instances. The limits of detection (LODs) and limits of quantification (LOQs) were established as the concentrations of analyte that resulted in signal-to-noise ratios of 3 and 10, respectively. These values were obtained by processing solutions with decreasing concentrations of the analytes; before analyzing each solution, water was processed to confirm the absence of contaminants and/or memory effects. The LODs ranged from 0.005 to 0.25 µg mL^−1^, whereas the LOQs were in the 0.02–1.0 µg mL^−1^ interval. Finally, the precision was established through the successive injection of three replicates of standard solutions of the analytes. The relative standard deviations (RSDs) obtained were in the 2–11% range.

### 2.2. Sample Treatment

For sample treatment UAE was applied, using methanol as the extractive solvent. Portions of 25 mg of the homogenized samples were placed in glass vials. Then, 5 mL of methanol were added to the vials, and the resulting suspensions were first vortexed, and then placed in an ultrasonic bath for 5 min. The supernatant was separated from the solid residue and filtered. Finally, a portion of the extract was treated with 0.1% hydrochloric and injected into the chromatograph. No significant peaks were observed in the chromatograms obtained when the solid residues were further treated with a second 5-mL portion of methanol and subjected to the same extraction protocol.

The recoveries obtained under the proposed treatment were calculated by spiking a sample with known amounts of the analytes. The amount of each compound added to the sample was 0.25 µg g^−1^. In this study sample GCE-3 was used because according to the label, it contained a greater number of ingredients. The recoveries were calculated by comparing the increments of the peak areas for the analytes in the spiked samples with those obtained for standard solutions containing an equivalent concentration of each compound. The values obtained are listed in Table 3. As observed, the recoveries ranged from 95% to 106%.

In order to study the intra-day precision of the entire procedure, three portions of the samples were spiked with the analytes and processed consecutively; the inter-day precision was obtained from six replicates of the spiked samples processed on different days. The results are listed in Table 3. This table shows that the RSD values were of about the same order as those found for standard solutions of the analytes (Table 2).

### 2.3. Quantification of Terpenes in Dietary Supplements

**Figure 2:**
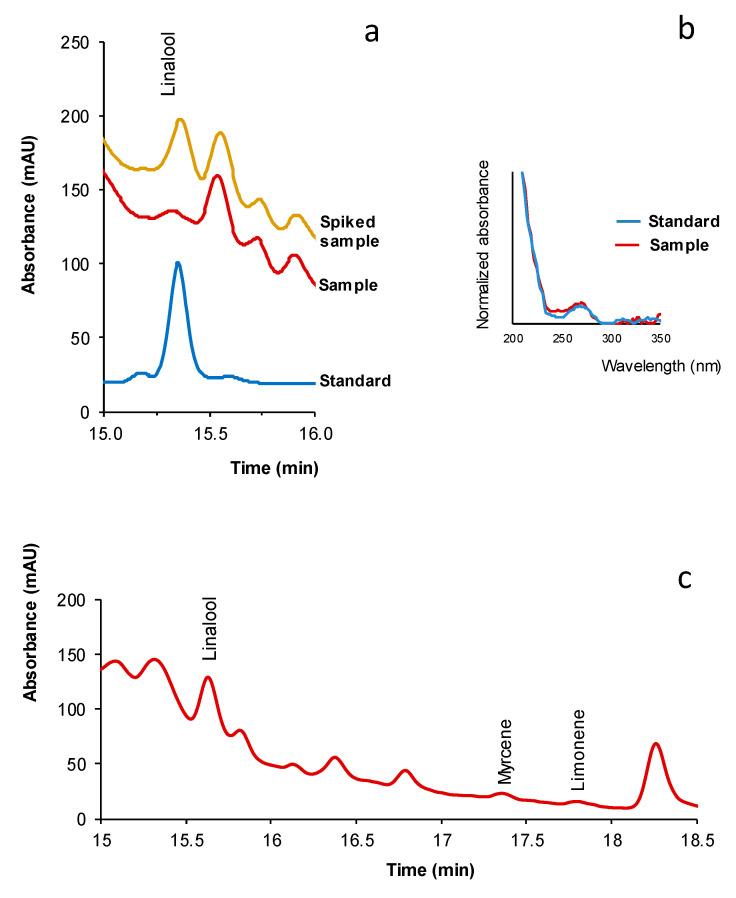
(**a**) Chromatograms obtained for a standard solution of linalool (5 µg mL^−1^), for the extract of sample FB-1, and the same extract spiked with linalool (1 µg mL^−1^). (**b**) normalized spectra of linalool and the peak attributed to linalool in the chromatogram of sample FB-1. (**c**) Chromatogram of the extract obtained for sample FB-2. For other experimental details, see text.

The proposed method was applied to the analysis of different dietary supplements, namely three green coffee extracts-based products claimed to enhance physical performance, two fat burning products for losing weight, and herbal preparation (relaxant). Portions of 25 mg of the samples were treated with 5 mL of methanol as described above, and then the extracts were chromatographed. The presence of a compound in a sample was established from the concordance between the retention times and spectra of the suspected peak and those of a standard solution, and it was further confirmed by spiking the extract with such a compound. This is illustrated in Figure 2a for the peak identified as linalool in the chromatogram corresponding to the extract obtained for sample FB-1. This figure shows the chromatograms of the extract obtained for the sample and the same extract spiked with 1 µg mL^−1^ of linalool; this figure also shows the chromatogram obtained for a standard solution of linalool. The normalized spectra registered for linalool in the standard solution and the peak attributed to linalool in the sample are shown in Figure 2b. A good correlation between the two normalized spectra can be observed. The chromatogram obtained for sample FB-2 is depicted in Figure 2c.

**Figure 3:**
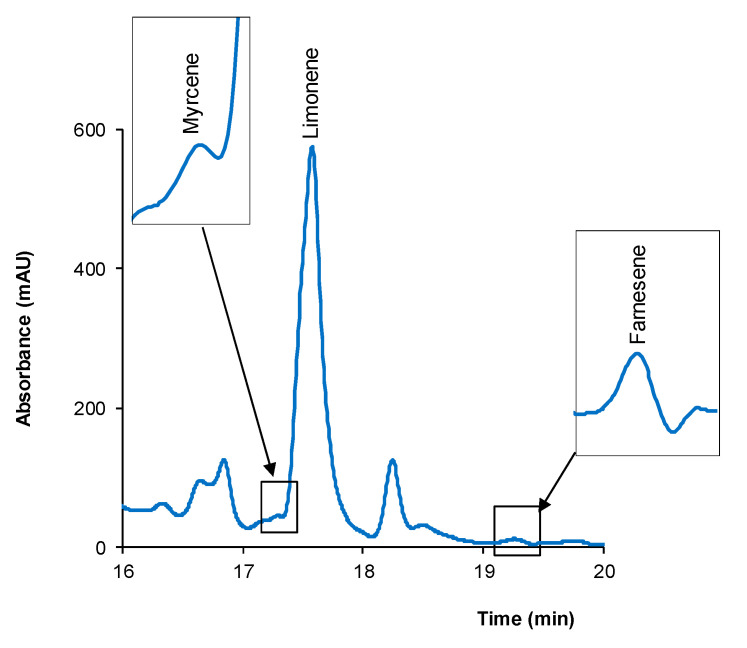
Peaks of limonene, myrcene, and farnesene in the chromatogram obtained for sample HP. For other experimental details, see text.

As an illustrative example of the signals of compounds present at very different concentrations, in Figure 3 are shown the peaks of the analytes found in sample HP for limonene, which corresponded to the highest concentration found for this compound throughout the study, and for farnesene and myrcene, both compounds present at the lowest concentrations in the sample.

For the quantitative study, the methanolic extracts obtained for each sample were chromatographed and the peak areas of the compounds found were calculated; if required, the extracts were previously diluted with water to adjust the peak areas of the analytes to their respective working linear intervals. The concentrations in the extracts were established from the calibration equations of Table 2, and then transformed into amounts of analyte in the samples taking into account the dilution factors (if applicable) and the recovery values of Table 3. The results obtained for all the samples tested are listed in Table 4.

The results of Table 4 indicate that all samples tested contained limonene and myrcene, except sample GCE-2. Linalool was found in the fat burning and relaxant preparations, although in the later sample its concentration was below its LOQ. α-Pinene was not detected in any of the samples assayed.

As it can be deduced from the results of Table 4, most compounds were present in the samples at sub mg per gram of product levels. Relatively high amounts of limonene were found in some of the samples; a high content of myrcene was found in sample GCE-3. In contrast, none of the tested compounds was found in sample GCE-2. Considering the rest of the samples, the lowest content of terpenes corresponded to sample FB-1, which contained a total amount of 0.136 mg of the tested compounds per unit. The highest value was found in sample HP, with 2.85 mg of the terpenes tested per unit. Expressed in relative terms, the highest amount of terpenes was observed in sample GCE-3, with a total amount of 4.28 mg per g of sample (0.482%). A high value was also found in sample HP (2.1 mg per g of product), whereas for the rest of the samples, the total amount of terpenes was 0.3–0.5 mg per g of sample.

## 3. Discussion

Several studies have been carried out to establish the content of relevant terpenes in different vegetal species and beverages for a better characterization of their flavor characteristics and/or to assess their biological activity [12,13,14,18]. What all those studies have in common is that GC is the technique used for the analysis of the samples. However, since LC is the dominant technique in laboratories dealing with the phytochemical analysis of medicinal plants and dietary supplements, it would be useful to have analytical alternatives based on LC [10].

**Figure 4:**
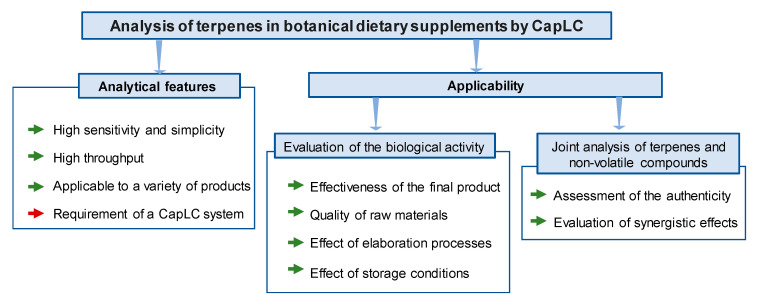
Analytical features and applicability of the method developed for the analysis of terpenes in botanical dietary supplements by CapLC; strengths in green; weaknesses in red.

In the present study, we have explored the potential of CapLC as an alternative to GC for the analysis of terpenic compounds in dietary supplements of botanical origin. Figure 4 summarizes the main conclusions of this study, expressed in terms of the advantages and drawbacks of the proposed approach.

First, from an analytical point of view, the proposed CapLC method allowed the quantification of the target compounds, even at sub mg per g levels, with suitable precision. The analytes were satisfactorily extracted from the samples by a simple UAE procedure. The high sensitivity and selectivity reached made unnecessary any preconcentration of purification steps; as a result, the whole analysis could be carried out in less than 30 min per sample. The selectivity was suitable so that the method could be applied to a variety of products that contained different botanical species as main ingredients. The requirement of a CapLC system is a limitation, although these kinds of systems are increasingly used in laboratories aimed at analyzing medicinal plants and dietary supplements. Moreover, unlike previous methods proposed for the analysis of terpenes in vegetal stuff, which in most instances involve MS detection, a simple UV detector is required.

As stated earlier, GC has been widely applied to the analysis of terpenes in plants and products elaborated from plants. However, in many cases, the real concentrations of the target compounds in the samples were not reported because the quantitative results were given as percentages of peak areas [15,17,19,20]. Kupska, et al. developed the GC x GC method with time-of-flight-MS detection to establish the terpenic profile of blue honeysuckle berries [13]. The volatile terpenes were previously isolated and concentrated by head-space solid-phase microextraction (HS-PSME). The concentrations measured in the sample extracts were lower than those measured in the present study. The concentrations of some terpenes, including linalool, in grapes, were measured by GC-MS in two studies. The target analytes were first extracted into a buffer, and then preconcentrated and purified by solid-phase extraction [14] or by HS-SPME [16]. Although the analytical performance of such methods was not reported, the concentrations of linalool measured were about the same order as those found in the present study (Table 4). Concentrations < 1 µg mL^−1^ of linalool were measured by He et al. in tea extracts by HS-SPME and GC-MS [18]. Compared with the proposed approach, the above results indicate that the concentrations of terpenes that can be measured by GC-MS are comparable or somewhat lower, although this can be partially attributed to the preconcentration achieved with the SPE or SPME treatments. The main advantages of the proposed CapLC method over GC-based assays are simplicity and speed.

As regards the utility, the proposed CapLC approach has been successfully used to establish the individual concentrations of representative terpenes in the methanolic extracts obtained from the samples assayed. Thus, the method can be applied to investigate the biological activity due to specific terpenes such as those included in the present study. It can be also used to compare the biological activities of different products through the calculation of the total amount of the selected terpenes. For example, from the results of Table 4, it can be deduced that the potential effects on health due to the presence of the selected terpenes in the products marketed as fat burnings are relatively low compared with the effects that can be expected for the other two types of products tested. Similar conclusions can be derived by comparing the contents of terpenes in products belonging to the same category. For example, the content of terpenes in sample GCE-3 was clearly superior to the amounts present in the other two products that contained green coffee extracts as the main ingredient; consequently, higher biological activity due to terpenes can be expected after the consumption of this product.

It must be remarked that none of the tested terpenes was found in sample GCE-2. The main ingredient of this product was decaffeinated green coffee extract, which suggests that the analytes were most probably lost during the industrial decaffeination process [28]. In contrast, sample HP contained four of the five terpenes included in the study, and a high amount of limonene; this is consistent with the fact that the manipulation of the ingredients involved in its production was clearly lower than that required to produce the other dietary supplements. Therefore, the proposed CapLC approach could be applied to evaluate the effect of the elaboration processes on the biological activity of the final products. It could be also used to estimate the sensory characteristics and the freshness of a product by determining the amounts of most volatile terpenes that remained in the sample after a period of storage.

Although in the present study the only compounds tested were terpenes, it has to be remarked that CapLC is a versatile technique that can be applied to the simultaneous determination of both volatile and non-volatile plant constituents. Thus, compounds with different physicochemical characteristics could be used as biomarkers to investigate the authenticity and safety of a given product in a single chromatographic run [11]. The technique could be also used to study possible synergistic effects on human health, which in many instances are not yet fully understood [6].

To summarize, CapLC is a versatile and useful tool that can be used to estimate the biological activity due to individual terpenes or a group of representative terpenes in dietary supplements elaborated from plant materials. The proposed method can be used as a reliable and versatile alternative to GC, and it can be easily implemented in laboratories dealing with the analysis of medicinal plants and dietary supplements, more familiarized with LC. The methodology used here offers advantages in terms of analytical performance and applicability, as can be deduced from Figure 4. The only limitation is the requirement of a CapLC system, although this may no longer be a problem considering that miniaturized techniques are increasingly used in laboratories devoted to the analysis of dietary supplements [23].

## 4. Materials and Methods

### 4.1. Chemicals and Solutions

All reagents used throughout the study were of analytical grade. Limonene, α-pinene, farnesene, myrcene, and linalool were obtained from Sigma-Aldrich (St. Louis, MO, USA). Hydrochloric acid (37%) was supplied by Scharlau (Barcelona, Spain). Methanol and acetonitrile (both HPLC grade) were purchased from VWR Chemicals (Randnor, PA, USA). Stock solutions of the analytes (1000 µg mL^−1^) were prepared by dissolving the appropriate amounts of the commercial standards in methanol. Working solutions of the analytes and their mixtures were prepared by diluting the stock solutions with water.

Ultrapure water was obtained from an Adrona system (Adrona, Riga, Latvia). Water was filtered through 0.22 µm nylon membranes purchased from GVS (Sandford, ME, USA) before use. All solutions were stored at 4 °C until use.

### 4.2. Chromatographic Conditions

The chromatographic system consisted of a capillary pump (Agilent 1100 Series, Waldbronn, Germany) equipped with a Rheodyne model 7725 six-port injection valve and a photodiode array detector (Agilent 1200 Series). An Agilent HPLC ChemStation system was used for data acquisition and calculation. A 15-cm fused silica capillary with an internal volume of 12 µL was used as an injection loop. Working solutions were loaded into the loop by means of a 25 µL precision syringe.

A Zorbax SB C18 (150 mm × 0.5 mm id, 5 µm) column (Agilent) was used for the separation of the target compounds. The mobile phase was a mixture of water-acetonitrile in gradient elution mode. The percentage of acetonitrile was linearly increased from 15% at zero min to 20% at 5 min, to 50% at 9 min, and to 75% at 11 min. Finally, the percentage of acetonitrile was increased to 100% at 15 min and kept constant until the end of the run. The mobile phase flow rate was 10 µL min^−1^. The analytical signal was recorded between 190 and 400 nm, and monitored at 200 nm unless otherwise stated.

The solvents were filtered through 0.22 µm nylon membranes (Teknokroma, Barcelona, Spain).

### 4.3. Analysis of Dietary Supplements

Different types of dietary supplements acquired in supermarkets located in the area of Valencia city (Spain) were analyzed: three green coffee extract-based products (GCE), two fat-burning formulations, and an orange herbal preparation. Sample GCE-1, marketed in the form of bags, contained unspecified amounts of *Carum carvi*, *Spiraea ulmaria*, *Paullinia cupana*, *Solidago virgaurea* L., *Foeniculum vulgare*, *Taraxacum dens leonis*, and *Coffea canephota*; the label declared the presence of limonene, although its concentration was not reported. The mean average mass of product per bag was 1.1 g. Sample GCE-2 contained 175 mg of decaffeinated green coffee extract (*Coffea arabica* L.) per capsule (0.40 g). Sample GCE-3 contained 200 mg of green coffee extract, 50 mg of green tea extract, 50 mg of *Citrus aurantium*, 10 mg of cayenne pepper, 12.5 mg of choline bitartrate, 12.5 mg of inositol, 0.7 mg of riboflavin, and 0.167 mg of chromium picolinate per capsule (mean average mass, 0.50 g). Sample FB-1 contained 125 mg of *Citrus aurantium,* 150 mg of *Raphanus sativus* L., and 125 mg of *Solidago Virgaurea* L. per capsule (0.50 g). Sample FB-2 contained 110 mg of *Citrus aurantium,* 55 mg of green tea, and 40 mg of cola nut extract per capsule (0.25 g). Finally, sample HP (1.40 g per bag) was a mixture of lime and leaves of orange trees intended marketed as a powder; linalool, α-pinene, and limonene were declared to be present, but their concentrations were not provided.

Accurately weighted portions of the samples (≈25 mg) were placed in 5 mL glass vials and treated with methanol. The mixture was vortexed for 1 min and then placed in an ultrasonic bath (300 W, 40 kHz, Sonitech, Guarnizo, Spain) for 5 min. Then the supernatants were removed and filtered with 0.22 µm nylon membranes. Finally, 90 µL of the extracts were placed in glass vials and mixed with 10 µL of a solution of 0.1% hydrochloric acid (*v/v*); if required, the extracts were previously diluted with water. Aliquots of the resulting mixtures (12 µL) were chromatographed.

All the experiments were carried out at room temperature.

## 5. Conclusions

In this work, we have applied for the first time CapLC to the determination of terpenes in dietary supplements elaborated with plant materials. The method offers high sensitivity and precision, making possible the quantification of the target compounds at concentrations ranging from 0.006 to 1.9 mg g^−1^. Compared with GC-MS methods reported previously, the UAE-CapLC method is less sensitive but much simpler and quicker. The potential utility of the proposed method has been demonstrated by comparing the individual and total amounts of selected terpenes present in dietary supplements as a simple way to assess their biological activities. Given the promising results obtained, future applications of this technique in the analysis of dietary supplements and medicinal plants can be expected.

## Figures and Tables

**Table 1:** Chemical structure and main effects of the tested compounds.

Compound	Structure	Activity
Linalool	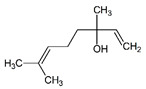	- Sedative [25] - Anti-inflammatory [8]
Myrcene	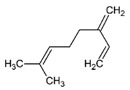	- Sedative [25] - Anti-inflammatory [8] - Geroprotective [9]
Limonene	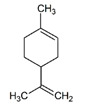	- Anti-inflammatory [8] - Antimutagenic [8] - Cardioprotective [9] - Antibacterial [9,12,13] - Geroprotective [9]
α-Pinene	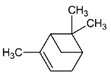	- Anti-inflammatory [8] - Antimutagenic [8] - Geroprotective [9] - Antibacterial [13]
Farnesene	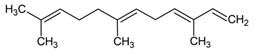	- Anti-inflammatory [8] - Antimutagenic [26]

**Table 2:** Times of retention (t_r_) and analytical parameters obtained with the proposed method.

Compound	t_r_ (min)	Concentration Interval (µg mL^−1^)	Linearity, n = 10 /R^2^	LOD ^1^ (µg mL^−1^)	LOQ ^2^ (µg mL^−1^)	RSD ^3^, n = 3 (%)
Linalool	15.5	0.02–1.0	y = (20,200 ± 200) x + (67 ± 90) /0.999	0.005	0.02	11
Myrcene	17.3	0.05–2.5	y = (530 ± 20) x +(−20 ± 20) /0.992	0.01	0.04	2
Limonene	17.6	1.0–10.0	y = (920 ± 40) x +(−600 ± 300) /0.993	0.25	1.0	3
α-Pinene	18.0	1.0–10.0	y = (166 ± 6) x +(−60 ± 30) /0.993	0.25	1.0	11
Farnesene	19.3	1.0–5.0	y = (900 ± 40) x +(−200 ± 100) /0.991	0.25	1.0	11

^1^ LOD—limit of detection; ^2^ LOQ—limit of quantification; ^3^ established at a concentration of 1 µg mL^−1^.

**Table 3:** Recovery and precision obtained in spiked green coffee. Values calculated for a spiked amount of the analytes of 0.25 mg g^−1^.

Compound	Mean Recovery (%)	Precision, RDS (%)	
		Intra-Day (n = 3)	Inter-Day (n = 6)
Linalool	96 ± 5	5	4
Myrcene	106 ± 3	3	10
Limonene	95 ± 10	11	13
α-Pinene	98 ± 9	10	13
Farnesene	105 ± 7	7	6

**Table 4:** Results obtained for the dietary supplements analyzed (n = 3).

Sample	Found Compounds	Found Amounts ^1^	
		mg per unit (n = 3)	mg per g (n = 6)
GCE-1	Limonene Myrcene	0.350 ± 0.004 0.059 ± 0.003	0.318 ± 0.003 0.054 ± 0.003
GCE-2	-	-	-
GCE-3	Limonene Myrcene	0.84 ± 0.01 1.3 ± 0.2	1.68 ± 0.01 2.6 ± 0.3
FB-1	Limonene Linalool Myrcene	0.129 ± 0.001 0.0031 ± 0.0001 0.0200 ± 0.001	0.258 ± 0.002 0.006 ± 0.001 0.039 ± 0.003
FB-2	Limonene Linalool Myrcene	0.130 ± 0.002 0.003 ± 0.001 0.0030 ± 0.0005	0.520 ± 0.005 0.012 ± 0.001 0.012 ± 0.001
HP	Limonene Linalool Myrcene Farnesene	2.60 ± 0.05 <LOQ 0.150 ± 0.001 0.100 ± 0.003	1.90 ± 0.04 <LOQ 0.110 ± 0.001 0.070 ± 0.002

^1^ Values expressed with digits known plus the first uncertain digit.

## Data Availability

The data presented in this study are available in this submission.
